# Role of SIRT3 in the regulation of redox balance during oral carcinogenesis

**DOI:** 10.1186/1476-4598-12-68

**Published:** 2013-06-23

**Authors:** I-Chieh Chen, Wei-Fan Chiang, Shyun-Yeu Liu, Pei-Fen Chen, Hung-Che Chiang

**Affiliations:** 1 Division of Environmental Health and Occupational Medicine, National Health, Research Institutes, No. 35, Keyan Road, Zhunan, Miaoli 35053, Taiwan; 2 Department of Oral & Maxillofacial Surgery, Chi-Mei Medical Center, Liouying, Tainan, Taiwan; 3 School of Dentistry, Yang-Ming University, Taipei, Taiwan; 4 National Environmental Health Research Center, National Health Research Institutes, Miaoli, Taiwan; 5 Department of Occupational Medicine, Taipei Medical University-Shuang Ho Hospital, Taipei, Taiwan

**Keywords:** Sirtuin 3, Reactive oxygen species, Oral squamous cell carcinoma, Human oral keratinocyte

## Abstract

**Background:**

Sirtuins (SIRT1-7) are a family of NAD-dependent deacetylases, which play an important role in regulating cancer tumorigenesis; however, their role in oral cancer has been controversial. SIRT3 is localized in the mitochondria, where it deacetylates and activates several enzymes involved in cellular redox balance and defense against oxidative damage.

**Results:**

We found that compared with normal human oral keratinocytes (HOK), SIRT3 is highly expressed in oral squamous cell carcinoma (OSCC) cell lines, but the enzymatic deacetylation is significantly reduced. We also sequenced the entire coding region of *SIRT3* and found the same mutation in 2 different OSCC cell lines. This point mutation is located in close proximity to the active site of deacetylase in the SIRT3 protein, and reduces the overall enzymatic efficiency of deacetylation. Furthermore, up-regulation of SIRT3 inhibited the cell growth of OSCCs and decreased the levels of basal reactive oxygen species (ROS) in both OSCC lines. To verify that the *SIRT3* sequence variation was associated with oral carcinogenesis, we sequenced the *SIRT3* gene from 21 OSCC patients, and 5 of the 21 patients (23.8%) carried the heterozygous missense mutation, p.Val208Ile. The heterozygous missense mutation in these patients was present in gremlin DNA isolated from both normal and tumor tissues.

**Conclusions:**

Our findings provide a valuable insight into the potential role of SIRT3 in the development of oral squamous cell carcinoma, by showing that a non-synonymous point mutation in *SIRT3* contributes to reduced catalytic activity of the protein and affects redox balance in OSCCs.

## Background

Oral cancer is the eighth most common cancer in men worldwide, and its incidence is increasing [1]. Oral squamous cell carcinoma (OSCC) accounts for >90% of oral cancers and can develop from oral precancerous lesions such as leukoplakias and erythroplakias [1,2]. A significant risk factor for the development of oral cancer is the chewing of areca nuts, which is a widespread habit throughout Southeast Asia [3,4]. Previous studies have revealed that areca nut extract (ANE) and other ingredients induce oxidative stress, elicit the formation of reactive oxygen species (ROS), and activate a stress response in cells [5-7]. ROS, such as superoxide (O_2_^-^) or hydrogen peroxide (H_2_O_2_), are constantly produced during metabolic processes in all living species. ROS can react with DNA, proteins, and lipids, and play important roles in many physiological and pathophysiological conditions, such as diabetes, neurodegenerative diseases, cancer, and aging [8]. Some chemical reactions involving ROS have considerable potential to damage genomic integrity [9]. The mitochondrial respiratory chain is one of the major sources of cellular ROS generation (~90% of ROS) [8]. In this cycle, electrons can escape the electron transport chain and react with molecular oxygen, leading to the generation of superoxides. ROS are involved in both the initiation and promotion of multistage carcinogenesis. Therefore, an understanding of mitochondrial regulation of ROS generation can provide important insights into the contribution of ROS to oral carcinogenesis. Recently, it was reported that several members of the sirtuin family (SIRT1-7), the human homologues of the Sir2 gene in yeast, play an important role in carcinogenesis [10]. The sirtuins are a family of nicotinamide adenine dinucleotide (NAD^+^)-dependent protein deacetylases [11], and their enzymatic activity is regulated by the ratio of NAD^+^ to NADH; high NAD^+^ levels activate sirtuins, and conversely, high NADH levels inhibit sirtuin activity [12]. Sirtuins regulate multiple cellular processes and physiological states, including the amount of oxidative stress, genomic stability, cell survival, development, metabolism, aging, and the longevity of organisms ranging from yeasts to humans [13-15]. Of the 7 SIRT analogues, SIRT1, SIRT6, and SIRT7 are primarily localized in the nucleus, whereas SIRT2 is in the cytoplasm, and SIRT3, SIRT4, and SIRT5 are localized in the mitochondria [16]. SIRT3 is synthesized as an inactive protein, is activated by matrix peptidase [17,18], and mediates regulation of oxidative stress [19-21]. In addition, SIRT3-deficient animals exhibit a striking global acetylation of mitochondrial proteins, while no significant changes in protein acetylation are observed in SIRT4^−/−^ and SIRT5^−/−^ mitochondria [22]. These findings indicate that SIRT3 is a major mitochondrial deacetylase. Acetylation by SIRT3 controls the enzymatic activity of mitochondrial proteins such as long-chain acyl-CoA dehydrogenase [19] in the fatty acid oxidation pathway, 3-hydroxy-3-methylglutaryl CoA synthase 2 in the ketone body synthesis pathway [23], ornithine transcarbamylase [24] in the urea cycle, isocitrate dehydrogenase 2 [21], and manganese superoxide dismutase [20,25] in the antioxidant system. Furthermore, the enzymatic activities of these enzymes are regulated by adaptive changes in acetylation in response to environmental stimuli [26]. In addition, a key role of SIRT3 in mitochondria is to serve as a mitochondrial localized tumor suppressor via its ability to inhibit mitochondrial ROS production [27-29]. Specifically, SIRT3 deficiency alone is sufficient to stimulate primary mouse embryo fibroblasts with *Myc* and/or *RAS* to form tumors in xenograft models. These data indicate that deletion of SIRT3 replaces the need for the loss of tumor suppressor required for transformation of primary cells by an oncogene. Additionally, studies have shown that germline SIRT3^−/−^ mice display increased levels of cellular ROS [20,21,27,30,31] and impaired cellular respiration in different tissues after prolonged fasting [19]. In contrast, SIRT3 overexpression *in vivo* suppresses cellular ROS levels [29]. In addition, these SIRT3^−/−^ mice display higher rates of high fat diet-induced obesity, insulin resistance, hyperlipidemia, and steatohepatitis [32]. The etiology of such defects may be found in the ability of SIRT3 to enhance cellular levels of antioxidants [19-21,24,33]. Although ROS levels were increased in SIRT3^−/−^ cells, these cells also contain detoxification enzymes that should scavenge the increased ROS. Thus, in accordance with the studies, cells lacking SIRT3 may have dysfunctional coordination of both mitochondrial respiratory chain and detoxification enzymes, which can result in aberrant and potentially damaging levels of ROS. Accordingly, this suggests that SIRT3 may regulate the initiation and progression of cancer by controlling the cellular redox balance. Although some investigators have suggested a particular role for SIRT3, these reports underscore the complexity of the biologic functions of SIRT3, which may differ according to the tissue of origin or cancer type. However, to our knowledge, the role of SIRT3 in regulating antioxidant defenses has not been investigated in oral squamous cell carcinoma. Therefore, the objective of the current study was to elucidate the role of SIRT3 in regulating cellular redox balance in OSCC.

## Results

### Variable levels of SIRT3 expression and its activity

**Figure 1:**
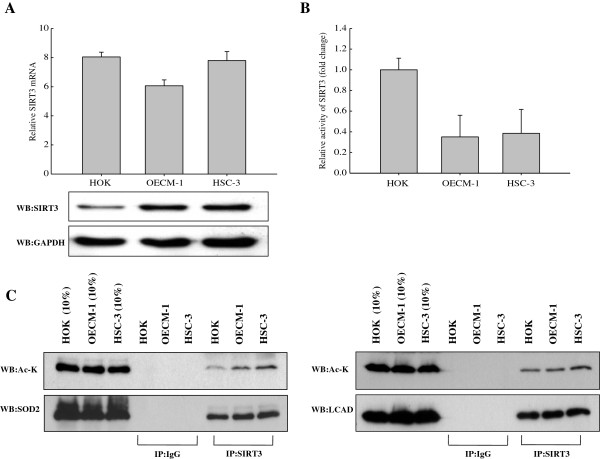
**Variable levels of SIRT3 expression and its activity are noted in normal cells (HOK) and OSCCs.** (**A**) RT-PCR and western blotting reveal the expression levels of SIRT3 in HOK and OSCC cell lines. (**B**) Specific activities of SIRT3 in HOK and OSCC cell lines were determined by enzyme assay. Each data point represents the mean ± SD from at least 3 independent experiments (mean ± SD). (**C**) Mitochondrial extracts from HOK, OECM1, and HSC3 cells were immunoprecipitated by using a SIRT3 antibody and analyzed by western blot using antibodies against SOD2, IDH2, and acetylated-lysine (Ac-K).

To examine whether expression of SIRT3 was different between normal primary human oral keratinocytes (HOK) and OSCCs, we examined the mRNA and protein levels in 2 OSCC cell lines (HSC-3 and OECM1) and compared those cells with HOK cells (Figure 1A). We found that SIRT3 was slightly overexpressed in both OSCC cell lines compared to expression in HOK, although the SIRT3 mRNA levels were lower in OSCC cell lines. To understand the function of SIRT3, we examined its enzymatic activity. For this purpose, we isolated the mitochondria fractions from HOK cells and OSCCs. Subsequently, endogenous SIRT3 was immunoprecipitated from mitochondrial fractions and tested for deacetylase activity. Surprisingly, we found that both OSCC cell lines had drastically lower levels of SIRT3 activity compared with HOK cells. The enzyme activities of SIRT3 in OECM-1 and HSC-3 were decreased by ~65% and 61%, respectively (Figure 1B). Because SIRT3 controls the enzymatic activity of mitochondrial proteins by deacetylation, we wanted to examine SIRT3-mediated deacetylation of its target proteins, such as long-chain acyl coenzyme A dehydrogenase (LCAD) in the fatty acid oxidation pathway, and manganese superoxide dismutase (SOD2) in the antioxidant system. We first examined the ability of SIRT3 to bind target proteins by co-immunoprecipitation. As shown in Figure 1C, western blotting detected SOD2 and LCAD in SIRT3 immunoprecipitates from mitochondrial extracts of normal cells and OSCCs. We next determined the acetylation levels of SOD2 and LCAD by western blotting, and found that the acetylation level of SOD2 was significantly lower in HOK cells compared with OSCCs. Furthermore, the acetylation level of LCAD was also slightly higher in OSCCs cultured under basal conditions. These results indicated that SIRT3 expression was slightly higher in OSCCs than in normal cells. However, the enzyme activity of SIRT3 was significantly reduced in OSCCs, leading to decreased deacetylation of mitochondrial proteins, which could be caused by a genetic change.

### Discovery of SIRT3 sequence variations

**Figure 2:**
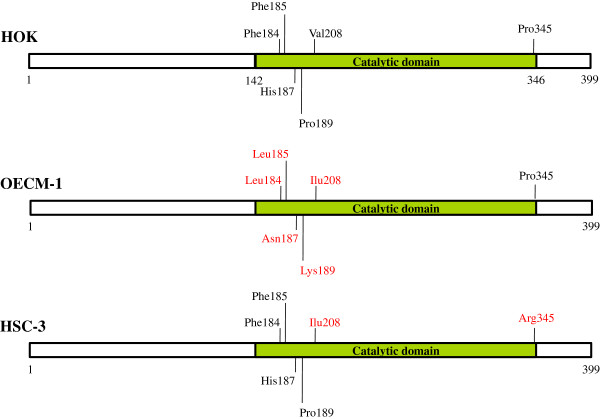
Schematic of SIRT3 protein and sequencing analyses identifying variations in the coding region of SIRT3 in HOK and OSCC cell lines.

**Figure 3:**
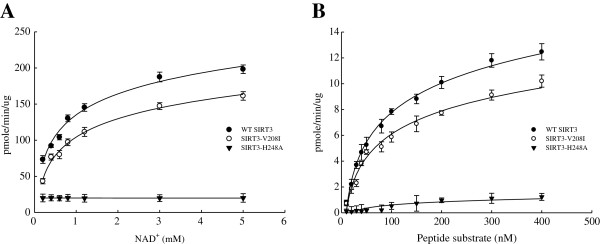
**Steady-state kinetic analyses of SIRT3 activity.** The rates of activity were measured as a function of NAD^+^ concentration (**A**) or peptide substrate concentration (**B**), as determined by fluorescence intensity of each sample. Each data point represents the mean ± SD from at least 3 independent experiments (mean ± SD).

Because the enzyme activity of SIRT3 was decreased in OSCCs, we tested whether sequence variations existed in the SIRT3 gene that could be correlated with decreased deacetylase activity in OSCCs. We sequenced the entire coding region of *SIRT3* in HOK cells and the 2 OSCC cell lines. From alignment of the SIRT3 gene DNA sequence in HOK cells and OSCC cell lines, we found 5 and 2 positions of genetic variation in the *SIRT3* coding region in OECM-1 and HSC-3 cells, respectively (Figure 2). As shown in Figure 2, these genetic variations in *SIRT3* were present within the conserved catalytic deacetylase domain of SIRT3 [34] and could therefore affect its enzymatic activity. Furthermore, the non-synonymous point mutation at nucleotide position 622, which led to the change of valine at position 208 to isoleucine (V208I), was present in both OECM-1 and HSC-3 cells. This change was located in close proximity to the deacetylase active site of SIRT3, and might be responsible for the reduced catalytic efficiency. To test for this possibility, we expressed recombinant WT SIRT3, SIRT3-V208I, SIRT3-P345R, and catalytically inactive SIRT3-H248A in *Escherichia coli* and tested their deacetylase activity *in vitro*. A steady-state kinetic analysis of SIRT3 activity was performed, and the initial rates of fluorescence release were measured as a function of NAD^+^ concentration. The resulting saturation curves were fitted to the Michaelis-Menten equation, and the V_max_ and *K*_*M*_ kinetic parameters were compared among WT SIRT3, SIRT3-V208I, SIRT3-P345R, and catalytically inactive SIRT3-H248A. We observed a 20% increase in the *K*_*M*_ value for NAD^+^ in SIRT3-V208I, compared to WT SIRT3, indicating more NAD^+^ was required for the SIRT3-V208I deacetylation reaction (Figure 3A and Table 1). Coincident with the increase in *K*_*M*_, we also observed a 19% reduction in the V_max_ for NAD^+^ in SIRT3-V208I, compared with WT SIRT3. In addition, we observed a 29% reduction in the SIRT3-V208I V_max_ for the peptide substrate, compared to WT SIRT3, but there was no change in the *K*_*M*_ for the peptide substrate (Figure 3B and Table 1). However, there was almost no affect on the enzyme activity of SIRT3-P345R (Table 1). These results suggest that in 3 independent enzyme preparations, the SIRT3-V208I mutation reduced catalytic efficiency by 37% compared to the catalytic efficiency of WT SIRT3 (Table 1). Together, these observations demonstrated that the mutant SIRT3-V208I had reduced enzyme efficiency, and this could partially explain why OSCC cell lines with the SIRT3-V208I mutation have lower SIRT3 activity than HOK cells.

**Table 1:** Summary of SIRT3 steady-state kinetic analyses

	**SIRT3**			
	**WT**	**V208I**	**P345R**	**H248Y**
**NAD**^**+**^**V**_**max**_**(pmole/min/mg)**	**16.03 ± 0.02**	**12.14 ± 0.06**	**15.94 ± 0.14**	**1.08 ± 0.16**
**K**_**M**_**(mM)**	**0.88 ± 0.01**	**1.07 ± 0.03**	**0.88 ± 0.07**	**N.D.**
**Catalytic efficiency (V**_**max**_**/K**_**M**_**)**	**18.21**	**11.35**	**18.11**	**N.D.**
**Peptide V**_**max**_**(pmole/min/mg)**	**17.5 ± 0.69**	**12.5 ± 0.03**	**17.32 ± 0.45**	**1.32 ± 0.11**
**K**_**M**_**(mM)**	**147.3 ± 12.4**	**153.4 ± 17.5**	**146.8 ± 10.8**	**54.2 ± 14.0**
**Catalytic efficiency (V**_**max**_**/K**_**M**_**)**	**0.11**	**0.07**	**0.12**	**0.02**

Rates of activity were measured as a function of NAD^+^ concentration or peptide substrate concentration, as measured by fluorescence intensity; n = 3 independent experiments (mean ± SD).

### SIRT3 gain of function regulates proliferation and ROS levels

**Figure 4:**
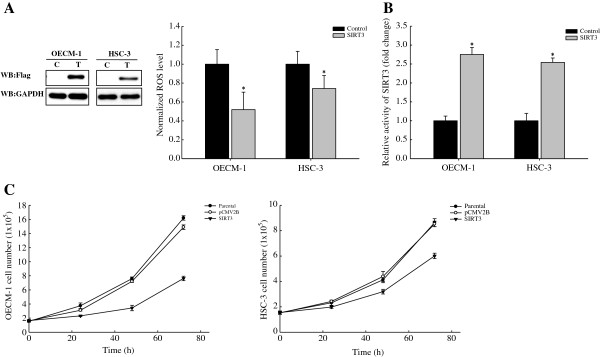
**ROS levels and cell proliferation are regulated by SIRT3 in OSCCs.** (**A**) Western blotting of whole cell lysates isolated from cells overexpressing flag-tagged SIRT3 and their relative fluorescence after incubation with DHE. C: control; T: transfected cells. (**B**) Relative activity of SIRT3 in OSCCs. OECM-1 and HSC-3 cell lines were transiently transfected with or without flag-tagged SIRT3. (**C**) Cell proliferation of SIRT3-overexpressing OSCC cell lines. Each data point represents the mean ± SD from at least 3 independent experiments (mean ± SD). The asterisk indicates a significant difference (*, p < 0.05) compared to control.

The *in vitro* kinetics assays demonstrated that recombinant SIRT3-V208I shows reduced enzymatic activity compared to WT SIRT3, indicating that the SIRT3-V208I mutant might contribute to the development of OSCCs. We thus sought to determine the effect of transiently expressed SIRT3 in OSCC cell lines by using a flag-tagged SIRT3 expressing vector. We observed a nearly 2- to 3-fold induction of flag-tagged SIRT3, relative to endogenous levels (Figure 4A). Furthermore, overexpression of SIRT3 decreased basal ROS levels by ~30% and 48% in HSC-3 and OECM-1, respectively (Figure 4A). We also confirmed the enzymatic activity of SIRT3 after transfection with expression vector. OSCC cell lines were transiently transfected with or without WT SIRT3, and SIRT3 activity was measured. Both the OECM-1 and HSC-3 overexpressing cell lines showed a mild increase in SIRT3 activity when transfected with the flag-tagged SIRT3-expressing vector (Figure 4B). Next, we examined whether SIRT3 had a role in cell proliferation by transiently expressing SIRT3 in OSCC cell lines. Exogenously expressed WT SIRT3 significantly decreased cell growth compared to the growth of vector transfected cells in both cell lines (Figure 4C). These data indicated that SIRT3 could possess tumor suppressor functions in OSCC cell lines, in addition to its previously described role in inhibiting transformation of primary cells [27].

### SIRT3 sequence variations in the OSCC patients

**Figure 5:**
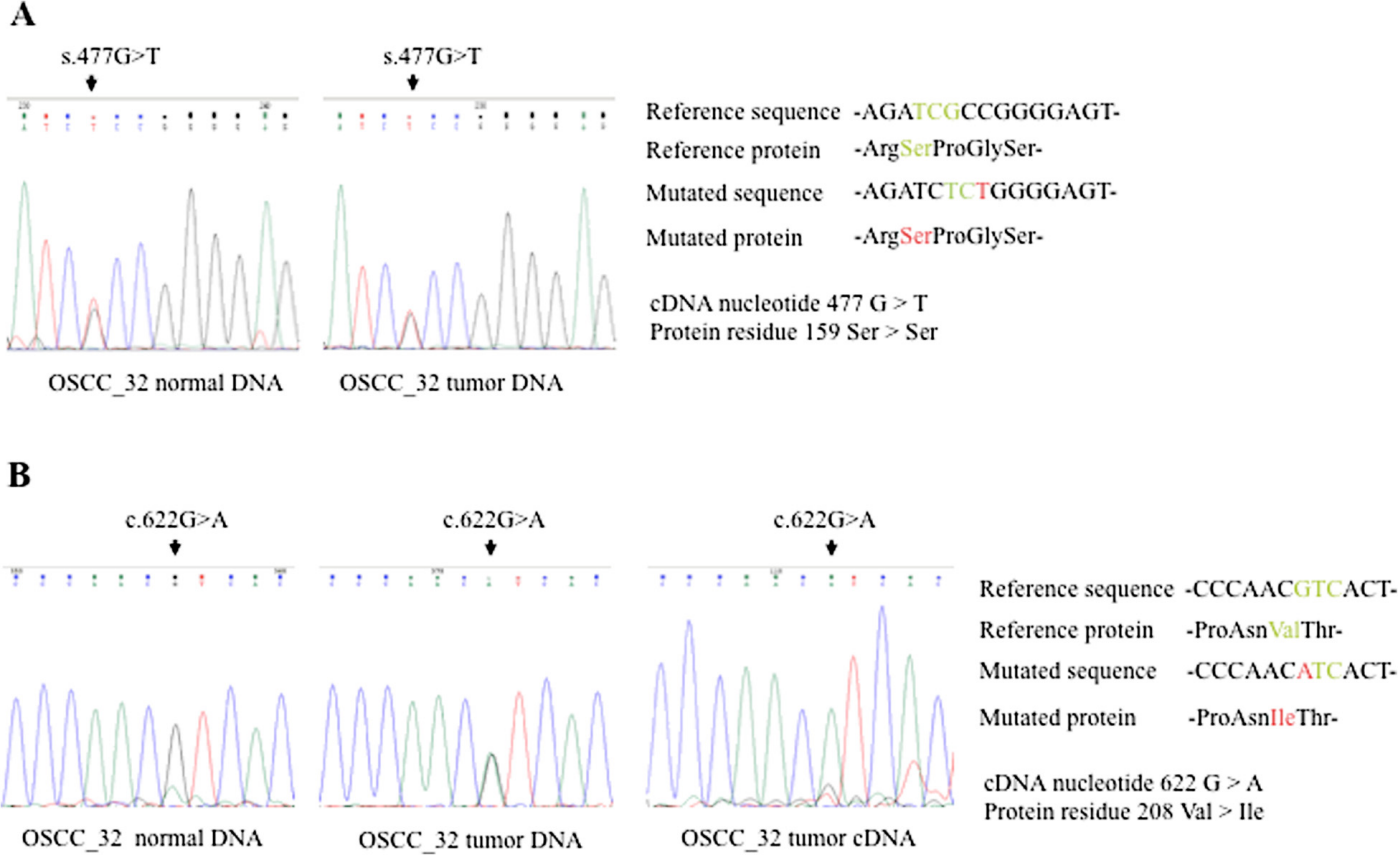
**Sequence chromatograms of DNA isolated from a patient with OSCC.** (**A**) Region harboring the s.477G > A mutation in patient OSCC_32. *Left*, heterozygous mutation in normal DNA; *Right*, heterozygous mutation in tumor DNA. (**B**) Region harboring the c.622G > A mutation (p.Val208Ile) carried by patient OSCC_32.*Left*, heterozygous mutation in normal DNA; *Middle*, heterozygous mutation in tumor DNA; *Right*, predominance of expression of the mutated allele in cDNA synthesized from OSCC_32 tumor RNA.

OSCC is a complex disease resulting from interactions between genetic and environmental factors [35,36]. Its development is a multistep process requiring the accumulation of multiple genetic alterations. These alterations are influenced by a patient’s genetic predisposition as well as by environmental factors, including tobacco, alcohol, chronic inflammation, and viral infection [35]. Because exogenously expressed WT SIRT3 produced inhibition of cell growth in OSCC cell lines, we tested whether variability in the SIRT3 gene was correlated with increased susceptibility for developing oral squamous cell carcinoma. We obtained reliable forward and reverse DNA sequencing results from amplified fragments of the SIRT3 gene obtained from 21 OSCC patients. We found that 4 of the 21 patients (19%, OSCC_31, 32, 39, 482) had a G to T transition at nucleotide 477 in exon 2 of SIRT3; this was a silent mutation that did not produce a change in the amino acid sequence. Another 5 patients (23.8%, OSCC_30, 31, 32, 39, 482) carried a G to A transition at nucleotide 622 in exon 3; this was a missense mutation resulting in the replacement of valine at residue 208 with isoleucine (p.Val208Ile). To validate our results and discriminate whether the detected SIRT3 mutations were present in the germ-line cells or somatic cells, we performed targeted exon sequencing of DNA isolated from tumor and normal cells of patients. Patients carried s.477G > T or c.622G > A as a heterozygous mutation in both normal and tumor cells. This finding indicated they had a germ-line genetic alteration of the SIRT3 gene (Figure 5), resulting in a V208I mutation in the mature protein. Sequence analysis of cDNA synthesized from tumor RNA revealed that the mutant allele was predominantly expressed in the tumor (Figure 5). Together, these results indicate that OSCC patients carried a germ-line mutation in the SIRT3 gene, represented by one single-base change, which introduced a non-synonymous point mutation that resulted in reduced catalytic activity of the SIRT3 protein. This could partially explain how patients with a V208I mutation in the SIRT3 enzyme may have an enhanced genetic susceptibility for developing OSCC.

### SIRT3 activity in OSCC patients

**Figure 6:**
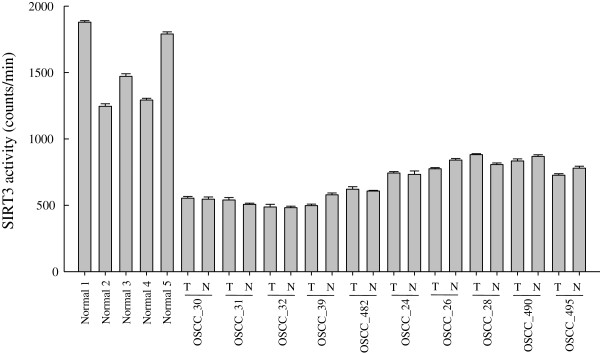
**SIRT3 enzyme activity of normal individuals and OSCC patients who did or did not carry the c.622G > A mutation of SIRT3.** N, normal tissue and T, tumor tissue. Each data point represents the mean ± SD from at least 3 independent experiments (mean ± SD).

A sequence analysis conducted with OSCC patients demonstrated that variability in the SIRT3 gene was correlated with increased susceptibility for developing oral squamous cell carcinoma. To obtain additional evidence for the effect of SIRT3 activity in OSCC patients, we analyzed the SIRT3 enzyme activity of normal subjects and OSCC patients. We collected 5 samples of normal gingival tissue from healthy individuals after tooth extractions, and 10 pairs of matched normal and tumor tissue samples from 21 OSCC patients. We then immunopurified endogenous SIRT3 proteins from these tissue samples and assayed for enzymatic activity. Surprisingly, we found that the SIRT3 proteins from tumor and normal tissues of patients (OSCC_30, 31, 32, 39, 482) who carried c.622G > A had drastically lower levels of SIRT3 activity compared tissues from normal individuals (Figure 6). The enzyme activity of SIRT3 from the 5 OSCC patients (OSCC_24, 26, 28, 490, 495) who didn’t carry this missense mutation was higher than activity in patients with the SIRT3 V208I mutation, but it remained lower than activity in normal individuals. These results indicate that the V208I mutation in the SIRT3 enzyme was capable of reducing the enzyme’s activity, and that decreasing SIRT3 activity may increase susceptibility to the development of oral squamous cell carcinoma.

## Discussion

In this study, we demonstrated that SIRT3 regulates cellular ROS, and this regulation has important implications for the growth of oral squamous cell carcinoma cells. It has been previously shown that SIRT3 contributes to cell survival by modulating oxidative stress pathways [37,38]. Our study showed that SIRT3 was slightly overexpressed in both OSCC cell lines compared with HOK cells, but the enzyme activity of SIRT3 was significantly reduced in OSCCs, which could be caused by genetic change. We found that increased activity SIRT3 in oral squamous cell carcinoma contributes to decreased ROS levels and increased cell proliferation. In addition, we sequenced the entire coding region of SIRT3 and found a non-synonymous point mutation at nucleotide position 622 in the SIRT3 coding region in both OSCC cell lines. Significantly, mutation of this position in the recombinant protein SIRT3-V208I was responsible for a 37% reduction of catalytic efficiency compared to WT SIRT3. This idea was further validated by the finding that in 5 of 21 OSCC patients, the SIRT3 mutation carried c.622G > A as a heterozygous mutation in both normal and tumor tissues, indicating that these patients had a germ-line genetic alteration of the SIRT3 gene. Additionally, we also determined the SIRT3 activity in 5 normal subjects and 10 OSCC patients (with or without c.622G > A) and found that all the OSCC patients had lower SIRT3 activity than normal subjects, and activity was especially lower in patients who carried the heterozygous missense mutation (c.622G > A). Taken together, these data indicate that SIRT3 may function as a tumor suppressor in OSCC cell lines by regulating cellular ROS levels and decreasing SIRT3 activity, and also may increase susceptibility to the development of oral squamous cell carcinoma. Our findings are consistent with previous work showing that SIRT3 functions as a tumor suppressor through regulation of ROS [27]. Kim et al. found that elevated ROS in the absence of SIRT3 increased genomic instability, promoting a tumor-permissive environment. We propose that SIRT3 might play a role in promoting tumorigenesis by altering cellular ROS levels.

In this study, we found that SIRT3 was slightly overexpressed in both OSCC cell lines compared with HOK cells, but the SIRT3 enzyme activity in OECM-1 and HSC-3 was decreased by ~65% and 61%, respectively. We sequenced the entire coding region of SIRT3 in HOK cells and the 2 OSCC cell lines, and found that the V208I mutation was located in close proximity to the deacetylase active site of SIRT3 in OECM-1 and HSC-3 cells. However, the enzyme activity of recombinant protein (SIRT3-V208I) was responsible for a ~37% reduction in catalytic efficiency compared to WT SIRT3. These data showed that overexpression of SIRT3 did not result in greater deacetylase activity in OSCCs, and that SIRT3 activity might be regulated by mechanisms such as post-translational modifications. Recently, it was discovered that SIRT3 is required for PGC-1α-mediated differentiation of brown adipose tissue in a manner that is dependent on the level of an estrogen-related receptor α (ERRα) [33,39]. The transcription coactivator PGC-1α could be regulating the SIRT3 gene involved in energy metabolism [40]. Thus, upon cellular stress, e.g., an increase in ROS or nutrient deprivation, human SIRT3 transcription might be stimulated [40], and the protein translocated to the mitochondrial inner membrane. There, SIRT3 could deacetylate and thereby activate the target enzymes and play a key role in modulating mitochondrial activities and the regulation of many pathways. However, current studies of SIRT3 have mainly focused on its cellular localization and identification of its substrates; the mechanism regulating SIRT3 function and activity remains unclear. We now know that the N-terminal extension of SIRT3 contains a mitochondria targeting signal peptide. During import of SIRT3 into the mitochondrial matrix, the protein is proteolytically cleaved at position 101 and thus enzymatically activated [18]. It has been postulated that the proteolytically shortened N-terminal region and the C-terminal extension form a module that might regulate access of substrate proteins to the active site [41]. Six phosphorylated serine residues (out of a total of 8 possible sites) between positions 101 and 118 may have been identified in a high-resolution mass spectrometry-based phosphoproteome analysis [42]. Therefore, it is possible that phosphorylation modulates the enzymatic activity of SIRT3 in mitochondria by regulating interaction of the N-terminal region with the catalytic domain. However, the biological relevance or influence on SIRT3 function has not yet been analyzed and more studies will be needed to understand the mechanism for regulation of SIRT3 activity.

In this study, we hypothesized that reduced SIRT3 activity might lead to an increase in cellular ROS signaling, thereby enhancing tumorigenesis. We identified the entire coding region of SIRT3 in HOK cells and 2 OSCC cell lines, and found a correlation between a genetic change at nucleotide position 622 in the SIRT3 gene in OECM-1 and HSC-3 cells. We tested for the possibility of a functional impact of the non-synonymous point mutation (V208I) in the SIRT3 protein. We used Ac­Arg­Gly­Lys(Ac)­AMC peptide as the substrate and determined deacetylase enzyme activity by measuring fluorescence intensity. As shown in Figure 3 and Table 1, the *K*_*M*_ value and V_max_ of SIRT3-V208I for NAD^+^ were 1.07±0.03 mM and 12.14±0.06 pmole/min/mg, respectively. The *K*_*M*_ v and V_max_ kinetic parameters were compared between WT SIRT3 and SIRT3-V208I, indicating a 20% increase in the *K*_*M*_ and a 19% reduction in the V_max_ for NAD^+^ in SIRT3-V208I. Moreover, we observed a 29% reduction in the SIRT3-V208I V_max_ for the peptide substrate compared to WT SIRT3, but there was no change in the *K*_*M*_ for the peptide substrate. These results indicate that the V208I lies within the conserved sirtuin catalytic deacetylase domain, and the mutation from valine to isoleucine reduced SIRT3 enzyme efficiency by both increasing the *K*_*M*_ for NAD^+^ and reducing the V_max_. Our data were consistent with a model in which reduction in SIRT3 enzymatic activity associated with the V208I mutation plays a pathogenic role in oral squamous cell carcinoma. Interestingly, it has been reported that genetic polymorphism in the SIRT3 gene is linked to longevity [43,44] and metabolic syndrome [32,45-48]. Similar results of V208I mutation had reported by Eric Verdin’s group [32] using different assay procedures. Verdin et al. used [^3^H-histone H4 peptide] as a substrate and measured rates of activity by using an organic-soluble radioactive signal. They found an 18% increase in the *K*_*M*_ and a 19% reduction in the V_max_ for NAD^+^ in SIRT3-V208I, compared to WT SIRT3, respectively. Their observations demonstrate that the SIRT3-V208I mutation reduced 34% catalytic efficiency and could increase susceptibility to developing the metabolic syndrome. However, less is currently known about the genetic polymorphism of SIRT3 in tumor cells. In the present study with 21 OSCC patients, we found that 4 (19%) had s.477G > T of SIRT3, a silent mutation producing no change in the amino acid sequence. Additionally, 5 patients (23.8%) carried c.622G > A of SIRT3, a missense mutation resulting in the replacement of valine at residue 208 with isoleucine (p.Val208Ile). As shown in Figure 5, the OSCC_32 patient carried either s.477G > T or c.622G > A as a heterozygous mutation in both normal and tumor tissues, indicating there was a germ-line genetic alteration of the SirT3 gene. The findings indicate that the SIRT3 +622A allele is positively associated with development of OSCC. This led to the reduction of catalytic efficiency in OSCCs, and promoted tumorigenesis by altering cellular ROS levels.

The exact role of mutant SIRT3 in tumorigenesis is poorly understood. Recently, several groups have provided evidence that SIRT3 is required to suppress tumorigenesis, and to induce stress-mediated cell death in tumors, including breast cancer, colorectal carcinoma, osteosarcoma, and leukemia [27-29,49,50]. Furthermore, Kim et al. showed that genetic deletion of SIRT3 pushes mouse embryonic fibroblasts in the direction of oncogenic transformation, and that SIRT3^−/−^ mouse embryonic fibroblasts exhibit stress-induced genomic instability [27]. While the activation of both oncogenes, *Myc* and *Ras*, is needed to transform an immortalized fibroblast into a tumor-forming cell, genetic deletion of SIRT3 reduced that requirement to only the activation of either *Myc* or *Ras*. Thus, SIRT3 functions as a tumor suppressor [51]. In addition, overexpression of SIRT3 is shown to decrease tumorigenesis in xenografts, even when induction of SIRT3 occurs after tumor ignition [29]. In our study, we determined the endogenous SIRT3 activity from 10 pairs of matched normal and tumor tissue samples obtained from OSCC patients. We found that the SIRT3 proteins from OSCC patients had significantly lower deacetylase activity than SIRT3 proteins from normal subjects, thus suggesting that a decrease of SIRT3 activity may increase susceptibility to tumor development. However, current studies of SIRT3 have mainly focused on its cellular localization and identification of its substrates; the molecular mechanism regulating SIRT3 activity remains unclear.

In this study, we began to investigate the potential role of SIRT3 in regulating cellular redox balance in oral squamous cell carcinoma and studied how SIRT3 is involved in oral carcinogenesis. However, the sample size of this study was small, and a larger number of OSCC patients must be evaluated for SIRT3 mutation to reach definitive conclusions about the role of this gene in the development of oral squamous cell carcinoma.

## Conclusions

In this study, we found that SIRT3 gene mutations were present in both OECM-1 and HSC-3 cell lines, and in OSCC patients, supporting the hypothesis that SIRT3 may act as a tumor suppressor and regulate the initiation and progression of cancer by controlling cellular redox balance. Our studies indicate that the germline mutation of the SIRT3 gene potentially plays a critical role in tumorigenesis and may contribute to increased susceptibility for the development of OSCC. Future studies screening a larger number of OSCC patients for SIRT3 mutations to highlight important genetic alterations may help identify useful targets for personalized cancer therapy and more successful cancer treatment.

## Methods

### Cell culture and reagents

HOK cells used in this study were cultured in oral keratinocyte growth medium (ScienCell, Carlsbad, CA, USA). Cells were cultured in a 37°C incubator filled with 5% CO_2_ and routinely passaged at 90% confluence. Two human OSCC cell lines, HSC-3 (tongue carcinoma), and OECM-1 (gingival carcinoma), were also used in this study. HSC-3 cells were cultured in Dulbecco’s modified Eagle’s medium with 2 mM glutamine; OECM-1 cells were maintained in RPMI 1640 medium. Each culture medium was supplemented with 10% fetal bovine serum and 100 units/mL of penicillin and streptomycin (Invitrogen, Camarillo, CA, USA). All OSCC cells were maintained at 37°C in a humidified atmosphere of 5% CO_2_.

### Genomic DNA, RNA isolation, and quantitative real-time PCR

For gene expression analysis, pairs of tumor and normal marginal tissues were obtained from 21 OSCCs. Frozen tissues were stored in liquid nitrogen at −196°C until use. Genomic DNA obtained from cultured cells and human tissue was subjected to bisulfite treatment using the EpiTect Bisulfite kit (Qiagen, Hilden, Germany) according to the manufacturer’s instructions. Total RNA obtained from cultured cells and human tissue was extracted using TRIzol reagent (Invitrogen). cDNA was then reverse-transcribed and amplified by PCR using a Transcriptor First Strand cDNA Synthesis kit (Roche Diagnostics, Mannheim, Germany). Quantitative RT-PCR was carried out using the FastStart Universal SYBR Green Master (Roche) on an Applied Biosystems ABI 7900 RealTime PCR System (Applied Biosystems, Foster City, CA, USA). The oligonucleotide primers for human SIRT3 and glyceraldehyde-3-phosphate dehydrogenase (GAPDH) were as follows: SIRT3-F 5′-ACCCAGTGGCATTCCAGAC-3′; SIRT3-R 5′-GGCTTGGGGTTGTGAAAGAAG-3′; GAPDH-F 5′-GAGTCAACGGATTTGGTCGT-3′; GAPDH-R 5′-GACAAGCTTCCCGTTCTCAG-3′. The gene expression level was normalized using GAPDH as an internal reference gene, and the average relative change was calculated using 3–5 determinations by relative quantification, applying the delta-delta cycle threshold method. This study was approved by the Institutional Review Board (IRB) of the Department of Oral and Maxillofacial Surgery of Chi-Mei Medical, Liouying, Taiwan (EC-1000202-R1).

### Plasmid construction and infection

The human SIRT3 coding region (GeneBank: NM_012239) was amplified by polymerase chain reaction (PCR) using the forward primer 5′-TTCGAACCATGGCGTTCTGGGGTTGG-3′, which introduced a Nsp V site, and 5′-CTCGAGCTATTTGTCTGGTCCATCAAGCTTCCC-3′, which introduced a XhoI site, under the following conditions: denaturing for 30 sec at 94°C, annealing for 30 sec at 62°C, and elongation for 1 min at 72°C for 35 cycles. The full-length of SIRT3 was subcloned into the constitutive mammalian expression vector, pCMV-Tag 2B (Stratagene, Amsterdam, Netherlands), and verified by DNA sequencing. Transfected cells were seeded in 6-cm cell dishes at 5 × 10^5^ cells/dish and transfected with pCMV2B-SIRT3 or empty vector using Lipofectamine reagent (Invitrogen), according to the manufacturer’s protocol. Transfected cells were used for further cell proliferation assays.

### Cell proliferation assay

Transfected cells were seeded in 96-well culture plates at 1 × 10^4^ cells/well and incubated for 72 h. Cell proliferation was assessed using the MTT assay according to the manufacturer’s recommendations (Roche).

### Mitochondria isolation and immunoprecipitation

Mitochondria were isolated from cultured cells by using a mitochondria isolation kit (Pierce Biotechnology, Inc., Rockford, IL, USA) following the manufacturer’s protocol. Isolated mitochondria were lysed with RIPA buffer, and then underwent direct western blot analysis or immunoprecipitation. Then, 2 mg of protein from samples (total lysates or mitochondrial extracts) was used for immunoprecipitation with a Pierce® Crosslink IP Kit (Pierce) following the manufacturer’s protocol, and analyzed by western blot.

### Western blot analysis

Cells were lysed directly in RIPA buffer and adjusted for protein concentration using the BCA protein assay kit (Bio-Rad, Hercules, CA, USA). Lysates were resolved by 10% SDS-PAGE and then transferred to PVDF membranes. The membranes were blocked and incubated with specific antibodies against SIRT3 (Cell Signaling), actin (Sigma-Aldrich, St. Louis, MO, USA), GAPDH (Santa Cruz Biotechnology, Santa Cruz, CA, USA), SOD2 (Epitomics, San Diego, CA, USA), LCAD (Pierce), and acetylated-lysine (Cell Signaling) antibodies. Proteins were visualized by enhanced chemiluminescence using an ECL-Plus detection system (Perkin Elmer-NEN, Courtaboeuf, France).

### ROS measurement

Cellular ROS was detected using the fluorescent dye dihydroethidium (DHE) obtained from Vigorous (Vigorous, Beijing, China) according to a previous study [52]. Cells were transiently transfected with or without flag-tagged SIRT3 and cultured in 6-well plates for 24 h. The cells were then washed with PBS and labeled with DHE (5 μmol/L dissolved in 1% DMSO) in the culture plates at 37°C for 30 minutes in PBS. Culture plates were placed on ice to stop the labeling, trypsinized, and resuspended in ice-cold PBS. Samples were analyzed using a flow cytometer (BD FACS Calibur, BD Biosciences, San Jose, CA, USA). The mean fluorescence intensity (MFI) of 10,000 cells was analyzed in each sample and corrected for auto-fluorescence from unlabeled cells.

### Enzyme activity assay

SIRT3 proteins from total lysates of cultured cells and human tissue were concentrated using a Pierce® Crosslink IP Kit (Pierce), according to the manufacturer’s recommendations. Protein concentration was determined using the Bio-Rad protein assay kit (Bio-Rad). The enzyme activity assay for SIRT3 was performed in 50 μL of deacetylase buffer (4 mM, MgCl_2_, 0.2 mM dithiothreitol, 50 mM Tris–HCl, pH 8.5) containing 25 μL of SIRT3 proteins (10 ng/μL), 2 mM NAD^+^, and 25 μL of 1 mM fluorogenic peptide substrate Ac­Arg­Gly­Lys(Ac)­AMC (R&D systems, Minneapolis, MN, USA). Deacetylation reactions were conducted at 37°C for 30 minutes, and stopped by adding 50 μL of stop solution made by combining recombinant mouse trypsin 3/PRSS3 AMC (R&D systems) and nicotinamide (Sigma-Aldrich) to final concentrations of 0.2 ng/μL and 4 mM, respectively. The assays were then incubated at room temperature for 15 minutes, and read at excitation and emission wavelengths of 380 nm and 460 nm, respectively in endpoint mode. The activity was measured with a SpectraMax M2 microplate reader (Molecular Devices Corporation, Sunnyvale, CA, USA).

### Statistical analysis

The data are reported as the mean ± S.D. of at least 3 independent experiments. The P values for linear trend of mRNA expression levels were analyzed using the t test (slope estimate) in simple linear regression models. The difference was considered statistically significant at the level of P < 0.05 or P < 0.01.

### Consent

Written informed consent was obtained from the patient for publication of this report and any accompanying images.

## Abbreviations

SIRT: Sirtuin; HOK: Human oral keratinocyte; OSCC: Oral squamous cell carcinoma; ROS: Reactive oxygen species; H2O2: Hydrogen peroxide; ANE: Areca nut extract; NAD: Nicotinamide adenine dinucleotide; NAM: Nicotinamide; GAPDH: Glyceraldehyde-3-phosphate dehydrogenase; DHE: Dihydroethidium.

## Competing interests

None of the authors of this manuscript had any conflict of interest regarding the study.

## Authors’ contributions

ICC carried out the gene expression analyses, DNA sequencing studies, enzyme activity assays, flow cytometry studies, participated in design of the study, and helped draft the manuscript. WFC examined the transfection study results and protein expression in the cell lines, and participated in designing the study. SYL carried out the flow cytometry studies, western blotting studies, and participated in designing the study. PFC conducted statistical analysis for the manuscript. HCC conceived the study, participated in its design and coordination, and helped to draft the manuscript. All authors read and approved the final manuscript.
